# Macroscopic superpositions and gravimetry with quantum magnetomechanics

**DOI:** 10.1038/srep37495

**Published:** 2016-11-21

**Authors:** Mattias T. Johnsson, Gavin K. Brennen, Jason Twamley

**Affiliations:** 1 Centre for Engineered Quantum Systems, Department of Physics and Astronomy, Macquarie University, Sydney, NSW 2109, Australia

## Abstract

Precision measurements of gravity can provide tests of fundamental physics and are of broad practical interest for metrology. We propose a scheme for absolute gravimetry using a quantum magnetomechanical system consisting of a magnetically trapped superconducting resonator whose motion is controlled and measured by a nearby RF-SQUID or flux qubit. By driving the mechanical massive resonator to be in a macroscopic superposition of two different heights our we predict that our interferometry protocol could, subject to systematic errors, achieve a gravimetric sensitivity of Δ*g*/*g* ~ 2.2 × 10^−10^ Hz^−1/2^, with a spatial resolution of a few nanometres. This sensitivity and spatial resolution exceeds the precision of current state of the art atom-interferometric and corner-cube gravimeters by more than an order of magnitude, and unlike classical superconducting interferometers produces an absolute rather than relative measurement of gravity. In addition, our scheme takes measurements at ~10 kHz, a region where the ambient vibrational noise spectrum is heavily suppressed compared the ~10 Hz region relevant for current cold atom gravimeters.

Gravimetry is the measurement of the local acceleration due to gravity on a test body. As gravity is an infinite range force that cannot be shielded against, it provides a useful probe for a variety of effects over a wide range of distances. Given a theory of gravity, gravimetry provides information about local mass distribution or, given a known, local mass distribution, it provides information that can be used test theories of gravity.

More specifically, precision gravimetry has numerous applications ranging from geophysics, geodesy and inertial sensing^1^,^2^,^3^,^4^, through to precision measurements of the fine-structure constant in quantum electrodynamics^5^ and the gravitational constant^6^,^7^,^8^. It has been used to test alternative theories of gravity and quantum gravity^9^,^10^,^11^, violations of local Lorentz invariance^12^,^13^ and has the potential to search for gravitational waves^14^,^15^. As with any scientific tool, as the precision of gravimetry increases, more systems and effects become amenable to gravitational analysis.

Gravimeters fall into two classes: relative and absolute^4^. Relative gravimeters measure gravity changes from some reference value, and thus require calibration against a known absolute value of gravity. Absolute gravimeters, on the other hand, do not require calibration, and provide an actual measurement of local gravity. The best relative gravimeters are more accurate at measuring changes in gravity than absolute gravimeters, but their calibration must be regularly reset, as all gravimeters are subject to both short and long-term drifts from their stable settings. In addition, their precision is only as good as the absolute gravimeter used for their calibration.

In this paper we consider absolute gravimetry. Current absolute gravimeters are based on acceleration measurements of freely falling objects, such as mirrored corner cubes or, more recently, ultra-cold atomic clouds^4^. As an example of what can be currently achieved, one of the most accurate commercially available absolute gravimeters is the Scintrex FG-5, which is based on a free falling corner cube combined with a Mach-Zehnder interferometer and atomic clock^16^. By utilising advanced vibration isolation techniques this instrument can achieve an absolute gravimetry precision of ~1.5 × 10^−7^ ms^−2^ Hz^−1/2^. Such an instrument is capable of detecting of large mineral deposits, and can measure changes that take place on a scale of days such as a large groundwater movement.

At a research level, however, over the last decade atom interferometers have exceeded the precision available from such falling corner-cube schemes. The initial implementation was carried out by Peters *et al.* and achieved a precision of 2.3 × 10^−7^ ms^−2^ Hz^−1/2 ^17,18. Since then there has been a great deal of progress in improving the technique^12^,^19^,^20^,^21^,^22^, with the current best precision reaching ~4.2 × 10^−8^ ms^−2^ Hz^−1/2 ^23.

One way to increase the precision of these atomic gravimeters further is to increase the free-fall distance of the atoms, which is currently on the order of a metre. Proposals have been made that extend the duration of the free-fall via micro-gravity/space based setups, and predict that such atomic gravimeters might reach precisions of ~10^−12^ ms^−2^ Hz^−1/2 ^24,25. If such schemes prove feasible, however, they will be most suitable for measuring static gravity or slow changes, and would be most sensitive to effects on a scale of many kilometers, as satellites are both rapidly moving as well as being hundreds of kilometers above the earth’s surface resulting in temporal and spatial averaging.

In this work we describe an entirely new form of absolute gravimeter that is Earth-based, compact, and does not rely on freely falling masses or atom interferometry. In contrast to existing methods, it is capable of measuring changes in gravity on the timescale of tens of microseconds and a spatial scale of nanometers. Our scheme performs absolute gravimetry measurements using a quantum magnetomechanical system consisting of a magnetically-trapped, superconducting micro-mechanical resonator whose motion is controlled and measured by a nearby RF-SQUID or flux qubit. The mechanical resonator is driven into a macroscopic quantum superposition of two different heights, which experience a relative energy difference due to their different heights in the local gravitational field. Over time this energy difference results in a relative phase difference which is then measured interferometrically via controlled gate operations on the flux qubit, resulting in a value for local gravity.

We show our scheme has the potential to achieve a gravimetry sensitivity of ~2.2 × 10^−9^ ms^−2^ Hz^−1/2^, which is over an order of magnitude better than the precision offered by the current state of the art absolute gravimeters. Furthermore, this value is limited only by the coherence time of the flux qubit, offering the possibility of significant improvements to the sensitivity in the future.

In addition, our scheme is based on engineering large spatial quantum superpositions of a massive object, which is of considerable interest beyond the gravimeter itself. Generating macroscopic quantum superpositions has been a much sought after goal both from the viewpoint of studying fundamental issues relating to the classical/quantum boundary as well as using such superpositions for other forms of enhanced sensing.

We note that while the predicted sensitivity of our scheme includes a number of loss and decoherence mechanisms, it does not include vibrational and other technical noise sources, and as such should perhaps be compared to the theoretical shot-noise limited sensitivity of an atom gravimeter, rather than what such devices can currently achieve. If we assume standard atom interferometer parameters, a one metre drop distance, and atomic clouds of 10^6^ atoms per shot, we obtain a fundamental sensitivity for an atom gravimeter of ~2 × 10^−9^ ms^−2^ Hz^−1/2^, similar to our scheme^26^. Note, however, that while vibrational noise from ~10–100 Hz is typically the limiting factor for atom interferometers^20^,^23^, this is likely to be much less of a problem for our scheme, as our single-shot operational time is of the order of ~100 microseconds.

The paper is structured as follows. We begin by describing the physical implementation of our scheme. We derive the trapping frequencies of the magnetically levitated resonator, and consider its stability. We derive the coupling between the resonator and the qubit we use to control and cool it, as well as describing the required physical properties of the qubit itself. Next, we describe the protocol used to carry out the gravimetry measurements, and detail the phase estimation scheme we use to translate the protocol’s interferometric result into a measurement of gravity. The use of this phase estimation scheme is crucial as at maximum sensitivity a fringe shift of ~10^9^ radians is obtained, and it is necessary to remove the associated modulo-2*π* phase abiguity. We then determine the maximum gravimeter sensitivity we can achieve with our scheme in the absence of decoherence, given the the physical contraints of the system such as the physical size of the qubit and resonator, as well as the coherence time of the qubit. Finally we consider the effect of realistic decoherence and noise factors on our gravimeter’s precision. We consider the imperfect preparation and readout of the qubit, as well the decoherence arising from damping and dephasing on both the resonator and the qubit. Some of the longer calculations and additional technical work can be found in the Appendices.

## Gravimeter Design

### Setup

**Figure 1:**
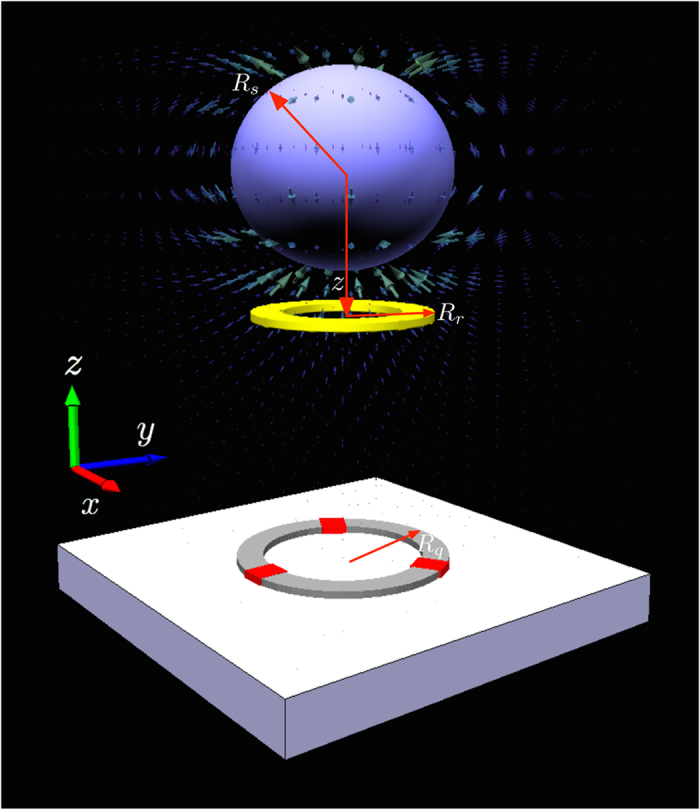
Schematic of the gravimeter setup. A small permanent magnet (blue sphere) with uniform magnetization in the upwards vertical direction produces a strong spatially inhomogeneous magnetic flux (shown as a vector field). A small superconducting ring (resonator - yellow), is trapped via the Meissner effect below the sphere. Currents in a flux qubit (grey) on a substrate (white) couple inductively to the motion of the trapped ring, so that the qubit can be used either to cool or coherently control the motion of the ring. The state of the qubit is readout using a DC SQUID - not shown.

As depicted in Fig. 1, we consider a small permanent magnet providing a highly spatially inhomogeneous magnetic field. We choose a sphere, radius *R*_*s*_, volume *V*, with uniform magnetization 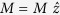, where 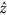 is oriented vertically upwards. We have found that 3D magnetomechanical trapping can occur with other shapes such as cones and spheres, but we choose the sphere for simplicity as the resulting fields, fluxes and potentials can be derived analytically.

Trapped a distance *z*_eq_ below the center of the sphere is a ring (the *resonator*), radius *R*_*r*_, of superconducting wire of thickness 2*a*, lying in the 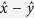 plane with self-inductance *L*_*r*_. As this resonator oscillates in the inhomogeneous magnetic field the magnetic flux it encloses changes, causing supercurrents to be generated within the ring to keep the overall enclosed flux Φ constant. The magnetic fields generated by these supercurrents will interact with the sphere’s field causing a mechanical restoring force on the resonator, trapping the resonator in all three directions^27^.

Located below the resonator is a superconducting flux qubit. The flux qubit generates counterpropagating supercurrents which can be in quantum superposition. These supercurrents generate magnetic fields and these couple via mutual inductance *M*_*rq*_ to the currents flowing in the resonator. With this coupling one can use the flux qubit to cool the motional state of the resonator^27^, and in addition one can use the qubit to coherently control the motion of the resonator^28^. We will use this latter capability to perform the interferometry protocol. The precision in the gravimetry protocol is directly related to how strong we can engineer this resonator-qubit coupling.

Provided the resonator is initially cooled to superconducting temperatures at some distance from the sphere, it will have zero magnetic flux threading it, and no persistent supercurrents. When it is moved into place below the sphere, supercurrents will be induced to ensure that it continues to enclose no flux. We work with a resonator that is the same size or larger than the flux qubit and is in very close proximity to it, ensuring that the qubit is shielded from any flux noise arising in the magnet.

To reduce damping loss of the resonator’s motion due to induced eddy currents in the spherical magnet we assume the magnet to be made from a magnetic insulator, e.g. Yttrium-Iron-Garnet (YIG), which possesses a saturation magnetization of *μ*_0_*M* ~ 0.17 T. Due to the resonator’s close proximity to the magnetised sphere and the fact that inductive coupling to the qubit results in large current densities, it must be composed of a superconductor with a high critical current and a high critical field. Further, to avoid decoherence due to flux pin dragging^29^, we require a Type-I rather than a Type-II superconductor. For these reasons we choose lead, which has a critical temperature *T*_*c*_ ~ 7 K and critical field *H*_*c*_ ~ 0.08 T, and limit the magnetisation of the sphere to this field strength.

Qubits are typically fashioned from either Aluminium or Niobium, with the latter having the advantage of a higher critical magnetic field. Specifically, the lower critical field for Niobium is between 0.2 and 0.3 Tesla, depending on fabrication^30^. It possible to construct a qubit as well as all the surrounding circuitry using only Niobium^31^, thus allowing the qubit to remain superconducting in the presence of the magnetic sphere.

### Oscillation frequencies and stability

As we intend to place the magnetically trapped resonator into a superposition of different vertical heights and let then them oscillate about the trapping minimum, it is necessary to determine the mechanical oscillation frequencies of the resonator. Since we want the resonator to only oscillate vertically in order to sample the gravitational field, we must also make certain that the resonator is also trapped in the horizontal direction, and that any horizontal oscillation modes have long periods compared to the vertical modes.

As shown in the Supplementary Appendix A, the resonator is trapped at a potential minimum below the sphere, and for small displacements undergoes harmonic oscillations in the vertical direction. The trapping arises from the Meissner effect, and the restoring force comes from supercurrents generated in the ring interacting with the magnetic field of the sphere as it moves away from the potential minimum.

Specifically, if the equilibrium vertical position point is at *z* = *z*_eq_, where *z* = 0 is the center of the magnetic sphere, then for small displacements the equation of motion for the resonator in the *z* direction is harmonic and is


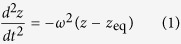


with the vertical oscillation frequency given by


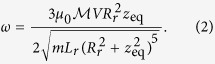


We also need to consider transverse trapping and oscillations firstly to establish that the resonator is indeed trapped in all three directions, and secondly to determine if there is any coupling between the vertical and horizontal motions. If this coupling exists then by cooling the vertical motion one cools the entire motion of the resonator, but such couplings can also lead to unwanted energy leakage from the coherent vertical dynamics to the transverse modes, leading to decoherence of our vertical superposition states.

As shown in the Supplementary A. to lowest order the trapping potential is given by


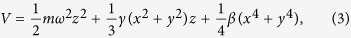


which describes a type of cross-mode coupling. For parameters described in Table 1, we find (*mω*^2^/2, *γ*, *β*) = (1.73 × 10^−2^ J, 1.98 × 10^3^ J m^−1^, 2.65 × 10^8^ J m^−2^).

The horizontal trapping at equilibrium (*z* = *z*_eq_) exhibits extremely slow oscillations. The period is amplitude-dependent, with higher amplitudes having shorter periods, but even with an unrealistically large amplitude of a 10 *μ*m the period is ~50 seconds, ensuring the horizontal dynamics are frozen out compared with the much fast vertical dynamics.

### Inductive coupling to the qubit

In order to force the resonator into a quantum superposition, as well as to cool it, we need to be able to coherently control it. This is accomplished through manipulating the state of the flux qubit, which is coupled to the resonator electromagnetically.

The strength of this coupling is determined by the mutual inductance between the currents flowing in the qubit and the small currents flowing in the resonator, the latter being dependent on the vertical position of the resonator. The coupling is of the form 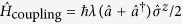, where 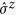 describes the direction of the current in the qubit, 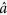, the annihilation operator for vertically trapped motional resonator phonons, and the coupling strength *λ* is defined as


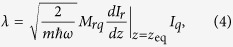


where *M*_*rq*_ is the mutual inductance between the resonator and the qubit, *I*_*q*_ is the current in the qubit, *dI*_*r*_/*dz* describes how the induced current in the resonator changes with respect to its vertical displacement from the equilibrium point *z*_eq_, and *m* is the mass of the resonator.

Using Eq. (2) and the expression for *dI*_*r*_/*dt* is derived in Supp Material A, and we find


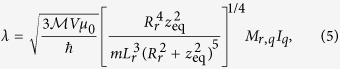


where *z*_eq_ is the vertical distance from the centre of the sphere to the resonator.

**Figure 2:**
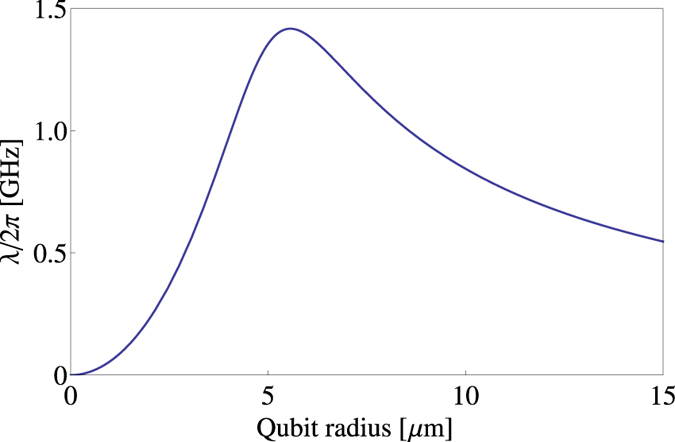
Value of the inductive coupling strength *λ* between the resonator and qubit for a fixed resonator radius of 5 *μ*m and a fixed sphere radius of 10 *μ*m as we vary the qubit radius. Maximal *λ* requires *R*_*r*_ = *R*_*q*_. System parameters as given in Table 1.

We want to make *λ* as large as possible, as this governs how quickly we can cool the resonator to its center of mass motional ground state, as well as how large we can make the resonator’s quantum superposition. Maximising *λ* requires the size of the qubit and resonator to be near identical 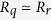. Assuming both are circular we obtain the results shown in Fig. 2. In addition, provided that we have control over the radius of the magnetic sphere, the maximal value for *λ* is obtained when the radius of the sphere is twice the radius of the qubit and resonator, i.e. *R*_sphere_ = 2*R*_*q*_ = 2*R*_*r*_. We assume these parameters thoughout the rest of the paper.

### Qubit subsystem

To cool and control the state of the resonator we use a flux qubit, which is a superconducting loop containing a Josephson-junction (JJ). The flux qubit is controlled by a magnetic flux which is generated by an external circuit.

To operate a flux qubit (or similarly an rf-SQUID)^32^, one applies a controlled external magnetic flux bias to yield an effective double-welled potential for the Hamiltonian of the qubit whose lowest energy symmetric/antisymmetric wave functions act as a two level system. These states correspond to oppositely circulating currents in the qubit loop and are split in energy depending on the height of the double well tunnel barrier. This splitting is quantified by the quantity *υ* = *L*/*L*_*J*_ − 1, where *L* is the geometric inductance of the qubit, and *L*_*J*_ is the Josephson inductance. Roughly speaking, *L*_*J*_ is controlled by the size and thickness of the junction, while *L* is set by the size and shape of the qubit loop. The energy splitting between the two levels increases as *υ* → 0, which means we need *L* ~ *L*_*J*_ in order to operate in a regime where the two qubit levels are sufficiently split in energy.

**Figure 3:**
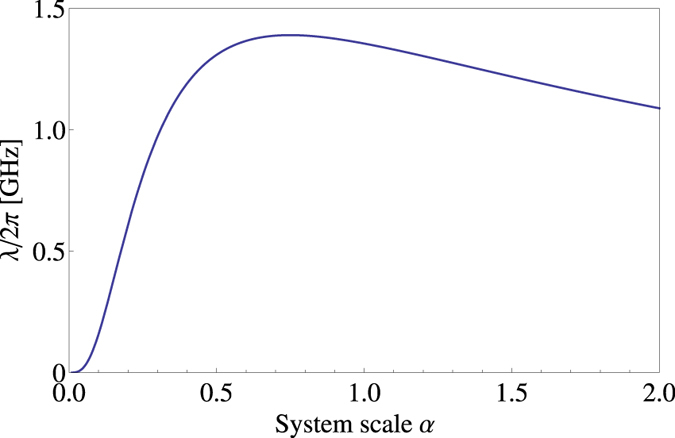
Behaviour of the inductive coupling strength between the motion of the resonator and qubit *λ*, as a function of overall system size, assuming the largest current that can be achieved using a flux qubit of a specific size. We take *R*_*r*_ = *R*_*q*_ = 5*α* *μ*m, *R*_sphere_ = 10*α* *μ*m, *a* = 1.0*α* *μ*m. Static parameters: *d* = 2 *μ*m, *r*_0_ = 1 *μ*m (see Table 1).

Since the inductance of a circular wire loop of radius *R* is roughly proportional to *R* ln (*R*), one cannot engineer very large qubit loops while still retaining the relation *L* ~ *L*_*J*_. As the inductive coupling also is proportional to the current in the qubit we require this also to be large but this is in conflict with large loop area as *I*_*max*_ ~ Φ_0_/2*L*. Thus there is a trade-off between the maximal current and physical size of the qubit — each contributing to the overall inductive coupling. For definiteness one can study the resonator-qubit inductive coupling strength *λ* as one scales up the physical size of the qubit/resonator/magnet (see Fig. 3), and curiously the optimal scale yielding the largest coupling strength is achieved when the flux qubit circular loop is quite small with radius ~5–10 *μ*m.

We note that in order to put our resonator into a cat state and use it as a gravimeter, it is necessary to ensure that we can begin with it in the motional ground state. This in turn requires that we have mechanism to cool it from its initial non-equilibrium state to the ground state by removing energy. Our cooling scheme is described in Supp Material B, and works by exploiting the strong coupling between the qubit and the resonator. We find that even with initial phonon occupation numbers as high as 10^9^ we can cool the resonator to the ground state, with an average final occupation number well below one.

## Gravimetry Protocol

### Description of gravimeter interferometry protocol

Before starting the protocol one must prepare the resonator in its trapped state and in the ground state of vertical motion as described above. We arrange, via tuning the frequency of the qubit for instance, to turn off the resonator-qubit coupling and to initialize the qubit in the state 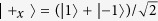. Next the resonator-qubit coupling is turned on. Notice that the effect of the qubit in ±1 eigenstates of 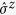, |±1〉, is to apply slightly different constant forces on the resonator in the vertical direction. These forces cause slight displacements in the trapping potential of the resonator providing for spatial superposition states which evolve in state dependent traps displaced from each other in the vertical direction. We then let the resonator evolve in these state dependent traps and after a specific duration the resonator will return to its initial height (which we denote as “one slosh”). However because of the slight difference in heights of the two traps a phase difference will accrue and when the resonator returns to its original height one will obtain constructive/destructive interference. This interference can be probed by again quickly turning off the resonator-qubit coupling and by measuring the qubit along the 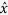-axis of its Bloch sphere. We will see that the phase difference will be directly proportional to the absolute acceleration due to gravity and we conclude with a rough estimate of the predicted sensitivty one might expect under a naive interferometric estimation protocol. In the following section we detail a more sophisticated estimation protocol that can yield far greater sensitivity and dynamic range in the gravimetry.

**Figure 4:**
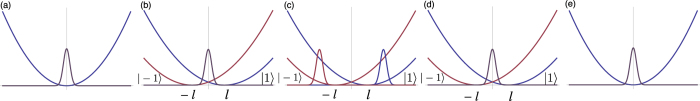
Illustration of the splitting protocol for gravimetry. Shown are the spin dependent harmonic trapping potentials along the 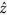 direction and the one dimensional spatial wave function for the resonator as a function of *z*. This protocol is repeated *M(K*, *k*) times for each value of the qubit resonator coupling *λ*_*k*_ = 2^*k*^*λ*_0_, for *k* = 0, … *K* where *λ*_0_ is a minimal value of the coupling. (**a**) At *t* < 0 the resonator is prepared in the ground motional state with an rms width *z*_0_ and frequency *ω*. The qubit is not coupled to the resonator (*λ*_*k*_ = 0). (**b**) At time *t* = 0 the qubit in prepared in the superposition state |+_*x*_〉 and the interaction *λ*_*k*_ is turned on. The trapping potential is now state dependent with minima located at ±*l* = ±*λ*_*k*_*z*_0_/*ω*. (**c**) After half an oscillation period, the state dependent motional wave packets are maximally separated by a distance 4*l*. (**d**) After a full oscillation period, the wave packets recombine, localised at the origin with the accumulated gravitationally induced phase *ϕ*_*k*_ mapped onto the qubit. (**e**) The interaction is turned off and the qubit is measured. For 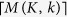 rounds the qubit is measured in the basis {|±_*x*_〉} basis and for the other 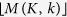 rounds in the basis 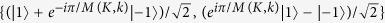 providing an estimate 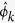 of the phase *ϕ*_*k*_. After the last stage the resonator returns to a ground state of the trapping potential.

We now use the protocol outlined above to measure absolute gravitational acceleration (shown diagramatically in Fig. 4).

As depicted in Fig. 4(a), we begin with the system in the state Ψ(*t* < 0) = |*α* = 0〉_*r*_ |+_*x*_〉_*q*_, where the subscripts *r* and *q* refer to the state of the resonator and qubit respectively, describing the resonator in a harmonic oscillator ground state, and the qubit in a superposition of counter circulating currents. At time *t* = 0 we apply the coupling Hamiltonian 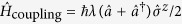, which imposes a constant force in the 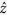-direction on the resonator depending on the qubit state. The full Hamiltonian of the system without a driving field on the qubit is


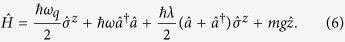


Rewriting in the position representation using 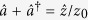 where 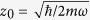 gives


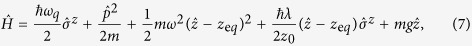


where *z* = *z*_eq_ is the equilibrium position of the resonator. Finally, completing the square and noting that 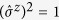 gives


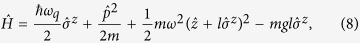


where we have dropped an additive constant, defined


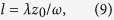


and shifted the origin of the *z* coordinate to the position *z*_eq_ − *g*/*ω*^2^. In this form, we see that because 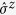 has eigenvalues ±1, we now have a double well potential, with the wells centred at ±*l* (see Fig. 4).

The resonator wave function now finds itself high on the harmonic potential slope, and experiences a state dependent force (see Fig. 4(b)). This means the wave packet will split into a superposition of two wave packets and each of these packets will oscillate in its state dependent trap *t* = *π*/*ω* (Fig. 4(c)).

We wait for the oscillation to complete (Fig. 4(d)), yielding the product state


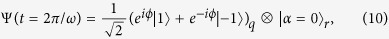


where the accumulated phase is


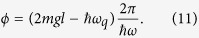


If we continue waiting, the system will undergo a series of *n* oscillations and following the rapid turn off of the coupling *λ* (Fig. 4(e)) we obtain the following reduced pure state for the qubit


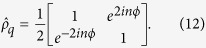


The expectation value for 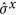 is


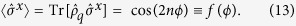


When functioning as a Ramsey interferometer, the phase sensitivity we obtain by measuring the state of the qubit is given by


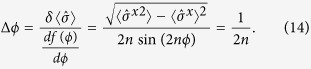


From (11) this means Δ*ϕ* = 1/2*n* = 4*πml*Δ*g*/*ħω*, where we have assumed precise knowledge of *ω*, *ω*_*q*_, *m*, *l* (methods to pre-determine these are discussed in Supp Material C, This gives an uncertainty in *g* of


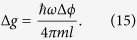


We note that *ω* scales as *m*^−1/2^, *l* scales as *λm*^−1/2^ *ω*^−3/2^, and *λ* scales as *m*^−1/4^. This means Δ*g* scales as *m*^−3/2^, indicating that all else being equal, the mass of the resonator should be as high as possible to maximize the sensitivity of the gravimeter.

### Phase estimation scheme

The main issue with the protocol as described so far is that when measuring the phase, we only get an answer modulo 2*π*, but the actual phase we care about is many times that, resulting a phase ambiguity. As an example, using the parameters in Table 1 we have a cat state separation of 2*l* = 1.9 nm, *ω* = 24.8 kHz and a resonator mass of *m* = 1.12 nanograms, so that one slosh takes *τ* = 2*π*/*ω* = 40.3 *μ*s and the accrued phase (after subtracting the known phase *ω*_*q*_*τ* accumulated due to the qubit splitting) is


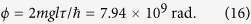


To solve the problem of phase ambiguity, rather than measuring *ϕ*, we choose to measure a much smaller phase, arising from a much smaller displacement of the resonator. Specifically, we choose a displacement small enough such that the phase *ϕ*_0_ we measure during the interferometric process is 0 ≤ *ϕ*_0_ < 2*π*. We then use the nonadaptive phase estimation scheme of ref. 33 to obtain this unambiguous phase with the same degree of precision as we would if we could measure the much larger phase *ϕ* without the 2*π* phase ambiguity.

The scheme works by determining *ϕ*_0_ via successive doublings of this phase, each providing another binary bit of precision to the final estimate. Doubling is achieved by doubling the current in the resonator, resulting in twice the resonator displacement. Each of the doubled phases is measured several times using the interferometric protocol described above with measurements of the qubit along specified directions along the equator of the Bloch sphere. Information from each of these measurements is used to refine the best estimate of the phase *ϕ*_0_ that has been obtained so far.

In detail, this works as follows: suppose we want to measure the phase *ϕ*_0_. We define *ϕ*_*k*_ = 2^*k*^*ϕ*_0_, with *k* = 0, 1, …, *K*. Then for each interferometric evolution with phase *ϕ*_*k*_ we make a measurement and repeat *M(K*, *k*) times where


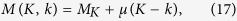


with *M*_*K*_, *μ* constants. Note that because the measurement protocol is non-adaptive, the measurements can be done in any order. For each round *k*, half (or a nearest integer thereof) of the *M(K*, *k*) measurements should be done in the qubit basis 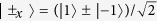 and the other half should be done in the 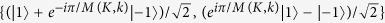 basis. At stage *k* = *K*, the phase is localised to an arc of size 2*π*/(3 × 2^*K*^) and the last estimate is used as the final estimate 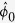.

We quantify the phase uncertainty by the square root of the Holevo variance^34^:


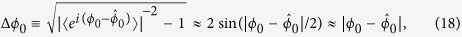


where the approximations hold when the variance is small. It is shown^33^ that the precision obtained with the nonadaptive measurement protocol with the choice *M*_*K*_ = 2 and *μ* = 3 provides a scaling of twice the Heisenberg limit (Specifically in ref. 33 they report a scaling of Δ*ϕ*_0_ < 2.03*π*/*N* for *K* = 20 doublings which improves for larger *K*.):


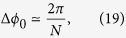


where


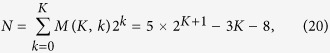


which is the cumulative accrued phase in units of *ϕ*_0_, during the complete estimation procedure. To ensure the phase *ϕ*_0_ is less than 2*π* we require *ϕ*_0_ = 2 *mgl*_0_*τ*/*ħ* < 2*π*, where 2*l*_0_ is the mean separation of the cat states, and *τ* is the interferometry *slosh* time, corresponding to one complete oscillation in the harmonic potential. Since *τ* = 2*π*/*ω* we obtain the condition


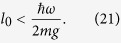


From Eq. (9) we see that


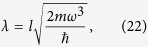


which means to get a displacement of *l* = *l*_0_ and an associated phase *ϕ* = *ϕ*_0_ we require a coupling strength of


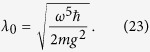


To obtain each of the doubled phases required for the protocol requires similar doubling of this coupling strength, i.e. *λ* = 2^*k*^*λ*_0_ is required to produce the phase *ϕ*_*k*_ = 2^*k*^*ϕ*_0_.

## Expected Sensitivity

Using Eqs (15) and (19) the gravimeter sensitivity obtained after one full cycle of the phase estimation scheme is


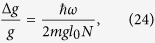


where *l*_0_ is the cat state displacement associated with a gravitational phase 0 ≤ *ϕ*_0_ < 2*π*, and *N*, the cumulative accrued phase over the entire cycle (in units of *ϕ*_0_). We note that through the use of this non-adaptive protocol one obtains a sensitivity that scales as *N*^−1^ rather than the usual *N*^−1/2^. Expressing *N* in terms of the upper doubling factor *K* using (20) yields


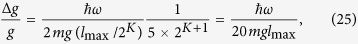


where *l*_max_ is the separation of the cat states corresponding to the maximum coupling strength *λ*_max_ = 2^*K*^*λ*_0_ generated. The value of the upper doubling factor *K* is determined by requiring that the *K*^*th*^ doubled fundamental phase 2^*K*^ *ϕ*_0_ is comparable with the overall accrued phase, i.e.


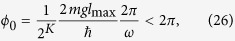


which means we need


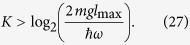


This is the precision we obtain after a single phase estimation cycle incorporating *N* projective qubit measurements. Each measurement involves initializing, evolving, and measuring the resonator over a fixed time duration which does not change throughout the cycle because we utilize a harmonic oscillator period that is constant irrespective of the spatial displacements of the wells. As we execute a complete *N*-measurement estimation cycle we only alter the double well displacements via *λ*_*k*_ but each of the *N* interferometry-measurement runs take the same duration of time. This permits us to quote an effective per-root-Hertz sensitivity if we repeat the entire estimation cycle many times.

In order obtain this per-root-Hertz sensitivity, we need to know how long this phase estimation cycle takes. First consider the time *τ*_exp_ for one interferometery run. This consists of: (i) a qubit reset time *τ*_reset_ to the |1〉 state, (ii) a single qubit rotation gate time *τ*_rot_ to the |+_*x*_〉 state, (iii) coherent evolution for one period of oscillation *τ*, (iv) single qubit rotation 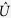 from either the |±_*x*_〉 basis or the 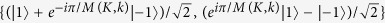 basis to the *σ*^*z*^ basis over a time *τ*_rot_, and finally (v) measurement of the qubit for a time *τ*_meas_. To obviate low frequency dephasing noise one could echo out noisy phases accumulated on off diagonal elements of the qubit state by inserting two additional steps between (iii) and (iv): (iiia) flipping the qubit state with a *σ*^*x*^ gate over a time *τ*_rot_, and (iiib) evolving the qubit for a time *τ* while decoupled from the oscillator, and then replacing 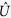 in step (iv) with the conjugated gate 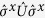. The total time for one run including the echo pulse is then


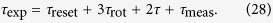


The total time required for one full phase estimation cycle is


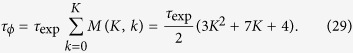


This means the per-root-Hertz sensitivity is


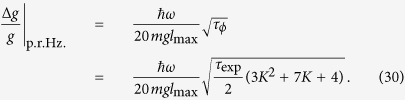


Using (27), in the *K* ≫ 1 limit we obtain


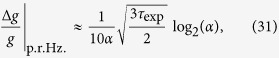


where


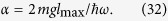


Thus the precision depends only on *α*, which should be as large as possible, which means engineering *λ* to be as large as possible. Lowering *ω* will also improve precision, but conflicts with our requirement that one slosh is completed within the coherence time of the qubit. Longer qubit coherence times would allow the precision to be improved. Finally, we want to make each preparation/slosh/measure sequence as quick as possible.

To obtain a quantative estimate of the precision our scheme can achieve, we assume the system parameters listed in Table 1. Using (21) with our assumed parameters we require *l*_0_ < 0.75 × 10^−18^ m. Thus we have *K* = log_2_ (*l*_max_/1_0_) = 30.2, indicating that we perform 31 doublings during the phase estimation protocol, increasing our initial phase from *ϕ*_0_ to 2^*K*^*ϕ*_0_.

**Figure 5:**
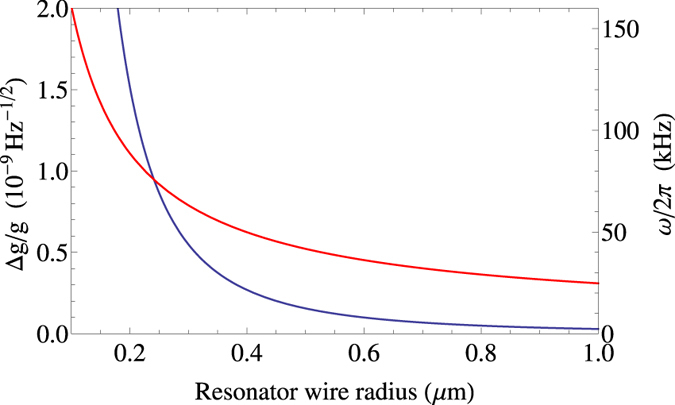
Mechanical oscillation frequency *ω* (red) and the per-root-Hertz sensitivity of our gravimeter in the absence of decoherence (blue) as a function of the resonator loop wire radius. Increasing the radius increases the mass of the resonator, which in turn reduces the oscillation frequency. Sensitivity increases rapidly as the thickness increases. This is due to a larger radius wire giving the resonator a larger mass and increasing the oscillation period. This in turn results in a strong coupling to the gravitational field, and a longer time spent sampling that field. System parameters are as given in Table 1.

We take thickness of the resonator loop wire as a free parameter to be chosen in fabrication. Changing this parameter has the effect of changing the mass of the resonator, which in turn alters the mechanical oscillation frequency. The frequency as a function of wire radius for our assumed system parameters is plotted as the red curve in Fig. 5. Changing the resonator mass will also change the precision, as it affects all the parameters *ω*, *λ*, *z*_0_, and *l*. The per-root-Hertz precision as a function of the resonator wire thickness is plotted as the blue curve in Fig. 5.

This sensitivity compares favourably with the best free-fall corner cube measurements (Δ*g*/*g* = 1.5 × 10^−8^ Hz^−1/2 ^16) and cold atom interferometers (Δ*g*/*g* = 4.2 × 10^−9^ Hz^−1/2 ^23).

We note that while our measurement protocol and phase estimation scheme gives us a phase, this phase must still be converted to a value for *g* via Eq. (11). Clearly, in order to obtain a precise estimate of *g*, we must know the parameters *m*, *ω*, *ω*_*q*_ and *λ* to the same level of precision. These quantities can be measured offline with any additional resources, and will not affect the time taken for the phase estimation protocol. We consider possible ways of accomplishing this in Supp Material C.

## Effect of Decoherence on Sensitivity

We now examine the effects of decoherence on the joint state of the resonator and the qubit as well as the effects of noise during the qubit preparation and readout stages. We assume a motional damping environment for the resonator and a damping and dephasing environment for the qubit. These are the dominant sources of decoherence in the system.

Other error processes include: noisy qubit initialisation in state |1〉_*Q*_, noisy implementation of a qubit unitary rotation 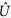, and imperfect measurement. Noisy initialization can be modelled as erroneously preparing (or resetting) a mixed input state by mixing in the complement to the ideal state with probability *p*_reset_ described by the map: 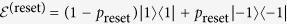. A noisy qubit rotation is modelled as a map where with probability 1 − *p*_rot_ the correct unitary opaerator 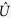 is applied and with probabiliy *p*_rot_ the qubit is completely depolarized: 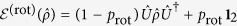, Noisy measurement is modelled as flipping the qubit with some probability *p*_meas_ before performing a perfect measurement: 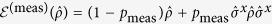. In a spin echo sequence, the qubit would be coupled to the resonator for a time *τ* = 2*π*/*ω* described by the map 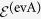, then the coupling would be set to zero, the qubit would be flipped with a 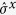 gate, and the system would freely evolve for a period *τ* described by the map 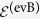. The composition of all these error processes in a full spin echo sequence gives a final output measurement of the desired value of cos (*ϕ*) of


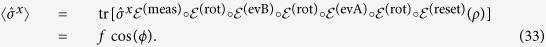


where we have introduced the cumulative per round fidelity


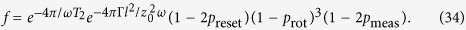


This form for the fidelity is valid when the rotation gate times and measurement times are small compared to the period of the resonator’s oscillation which is usually the case. If not then the factor 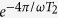 should be replaced by 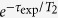.

As described in Supp Material D, the total decoherence factor *γ* after a single resonator oscillation is


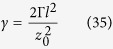


where Γ is the decoherence rate. For the system parameters in Table 1, the various contributions to motional decoherence of the resonator are Γ_rad_ = 3.3 × 10^−22^ s^−1^ (dipole radiation); Γ_eddy_ = 8.1 × 10^−19^ s^−1^ (eddy currents in sphere); and Γ_gas_ = 2.7 × 10^−8^ s^−1^ (background gas collisions). Using Eq. (35) yields the following decoherence factors after a single resonator oscillation with the maximum separation


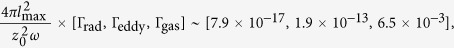


indicating that collisions with background gas molecules is the most significant form of amplitude damping, and even this gives a 99.4% fidelity after a single oscillation period.

Each stage *k* of our protocol involves estimating the value of the phase by estimating the probability the qubit is in state |*M*〉, i.e. an estimation of *p*_*M*_ = (1 ± cos (*ϕ*_*k*_))/2. Given the reduced polarisation of the qubit due to errors (Eq. 33), the procedure is akin to estimating the probability *p* that a biased coin lands heads subject to noise such that each observation gets flipped with probability *p*_noise_ = (1 − *f*)/2. This scenario was studied in ref. 35 where it was shown that a hedged maximum likelihood method provides a good estimate of an unknown *p* given a known *p*_noise_. The effect of the reduced visibility due to finite fidelity is to increase the number of measurements per stage, *M(k*, *K*) by a factor of 1/*f* ^2^ in order to keep the same overall sensitivity of our protocol. This multiplicative factor is independent of the stage *k* since the the operation time always involves single sloshes whose period is solely determined by the resonator frequency *ω*. The overall effect on the sensitivity is then


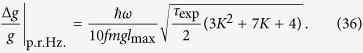


In order to determine the fidelity, we need to know qubit operation times, qubit error rates, and dephasing time. Recent experiments using superconducting transmon qubits in three dimensional microwave have shown dephasing times of 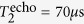, reset times *τ*_reset_ = 3 *μ*s and error rates *p*_reset_ ≤ 0.005^36^. All the other operations needed for fault tolerant quantum computation have been demonstrated with superconducting qubits as well. In ref. 37 the following operation times and errors were reported for transmon qubits: *τ*_rot_ = 40 ns, *τ*_meas_ = 4 *μ*s, *p*_rot_ ≤ 0.003, *p*_meas_ ≤ 0.09.

**Figure 6:**
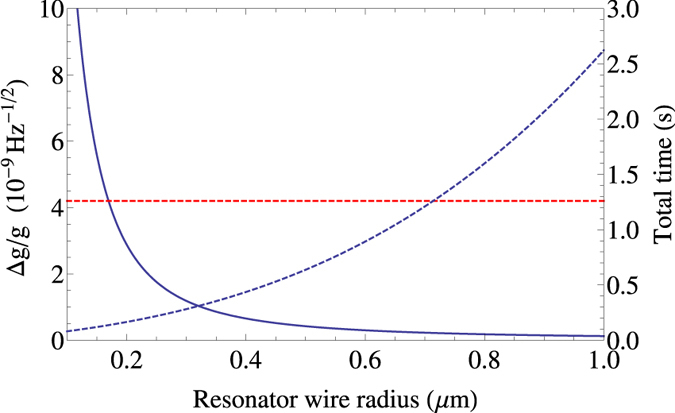
Performance of our gravimeter as a function of the resonator loop wire radius taking into account current experimental preparation, readout, and dephasing times. Solid blue line shows per-root-Hertz sensitivity; dashed blue line shows the time required for a full Δ*g*/*g* measurement, taking into account the requirement for more measurements as fidelity decreases; red dashed line indicates current best absolute gravimeter precision^23^. In principle sensitivity can be increased without limit, but at some point long term equipment drift or the timescale of the phenomenon of interest will become an issue. For that reason we take a 1 *μ*m resonator wire radius as a plausible upper limit. System parameters are as given in Table 1.

If we assume a flux qubit with these operation times, we can use Eq. (34) along with Eq. (28) to obtain *τ*_exp_. We can then use Eq. (36) to determine the ultimate sensitivity of our gravimeter, taking into account qubit errors, readout and preparation time and decoherence. The result is plotted in Fig. 6 showing for a resonator wire thickness of 1 *μ*m an achievable sensitivity of 
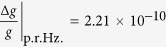
 Hz^−1/2^. Even with the decrease in fidelity as we increase resonator mass, the per-root-Hertz sensitivity still increases monotonically with this increase, albeit at a slower rate than the perfect decoherence-free case shown in Fig. 5. This sensitivity is over an order of magnitude better than the Δ*g*/*g* = 4.2 × 10^−9^ Hz^−1/2^ achieved by current state-of-the-art absolute gravimeters which rely on atom interferometry^23^.

## Conclusion

We have presented a scheme for absolute gravimetry utilising quantum magnetomechanics and Schrödinger cat states. The protocol interferometrically measures the differential gravitational phase accrued between the two heights of a macroscopic quantum resonator placed into a vertical spatial superposition. With realistic materials and current reported values for superconducting qubit coherence times we obtain a sensitivity of Δ*g*/*g* = 2.21 × 10^−10^ Hz^−1/2^ for the thickest resonator wire we considered, which is over an order of magnitude better than the Δ*g*/*g* = 4.2 × 10^−9^ Hz^−1/2^ achieved by current state-of-the-art absolute gravimeters which rely on atom interferometry^23^.

In addition to being more accurate than current methods, our scheme is capable of making gravimetry measurements on time and distance scales orders of magnitude faster and smaller than falling corner cube or atom interferometery-based gravimeters. A single shot measurement requires tens of microseconds to complete, compared to seconds for current methods, and the measurement takes place over a region of nanometers rather than meters. This allows the detection of transient gravitational changes in an entirely new regime, as well as allowing for operation of the gravimeter in a frequency range where vibrational noise is suppressed.

The sensitivity of our proposed gravimeter is constrained by the dynamic range of the qubit-resonator coupling parameter, as well as the coherence time of the qubit. The coupling strength is limited at the low end by the current noise floor of the qubit, and at the high end by the critical current value of the qubit and the inhomogeneity of the magnetic field of the sphere levitating the resonator. It is likely that this sensitivity can be substantially improved on, primarily by improving the coherence time of the flux qubit, but also by using lower temperatures and more complicated magnet-resonator geometries.

Finally, we note an intriguing application of precision gravimetry is to probe the role of gravity in quantum state reduction. Several models have been proposed which make predictions on the magnitude of gravitationally induced decoherence rates on macroscopic spatial quantum superpositions^38^,^39^. One model, due to Diósi^40^, predicts a decoherence rate *γ*_grav_ that scales with the difference between the gravitational interaction energy between the constituent separated mass distributions of a macroscopic superposition and the localized distributions. For the parameters considered in this work (summarized in Table 1), the predicted rate is *γ*_grav_ ~ 10^−9^ Hz which is on par with the magnitude of decoherence due to eddy currents induced in the magnetized sphere *γ*_eddy_ ~ 4.8 × 10^−9^ Hz. However, this is 11 orders of magnitude slower than the decoherence rate *γ*_gas_ due to background gas collisions so it would be extremely challenging to observe such effects with the current proposal and instead would likely require much larger sized Schrödinger Cat states.

## Additional Information

**How to cite this article**: Johnsson, M. T. *et al.* Macroscopic superpositions and gravimetry with quantum magnetomechanics. *Sci. Rep.* **6**, 37495; doi: 10.1038/srep37495 (2016).

**Publisher’s note:** Springer Nature remains neutral with regard to jurisdictional claims in published maps and institutional affiliations.

## Supplementary Material

Supplementary Information

## Figures and Tables

**Table 1:** System parameters and the values used in the main text for precision gravimetry.

Symbol	Value	Definition
Φ_0_	=2.07 × 10^−15^ Wb	flux quantum
*g*	=9.81 m s^−2^	acceleration due to gravity
*m*	=1.12 × 10^−12^ kg	resonator mass (Pb)
*ω*/2*π*	=24.8 kHz	resonator frequency
*z*_0_	=1.74 × 10^−14^ m	ground state rms width of resonator
*R*_*q*_	=5 *μ*m	radius of qubit loop
*R*_*r*_	=5 *μ*m	radius of resonator ring
*R*_sphere_	=10 *μ*m	radius of magnetized sphere
*a*	=1.0 *μ*m	radius of resonator wire
*d*	=2.0 *μ*m	distance between resonator centre of mass and qubit
*r*_0_	=1 *μ*m	minimum distance from sphere surface to centre of mass of resonator
*z*_eq_	=11 *μ*m	equilibrium position of resonator
*V*	=4.19 × 10^−15^ m^3^	volume of magnetised sphere
*M*	=8.76 × 10^2^ A m^−1^	magnetisation of YIG sphere
*ρ*	=10^12^ Ωm	resistivity of magnetised sphere made of YIG
*l*_max_	=9.5 × 10^−10^ m	largest size of Schrödinger cat
*λ*_max_/2*π*	=1.35 GHz	maximum qubit-resonator coupling
*λ*_0_/2*π*	=0.63 Hz	minimum qubit-resonator coupling
*L*_*r*_	=2.25 × 10^−11^ H	resonator self inductance
*L*_*q*_	=1.38 × 10^−11^ H	qubit self inductance
*M*_*rq*_	=6.75 × 10^−12^ H	mutual inductance between resonator and qubit
*ω*_*q*_/2*π*	=6 GHz	qubit energy level splitting
Φ	=2.37 × 10^−12^ Wb	flux through the resonator
*T*_*q*_	=100 mK	temperature of qubit system
*I*_*q*max_	=75 *μ*A	maximum current in qubit
*I*_*q*0_	=3.5 × 10^−14^ A	minimum current in qubit
*I*_*r*max_	=48 *μ*A	maximum current in resonator
*τ*_exp_	=87.8 *μ*s	time for one complete prepare/evolve/measure run
*τ*_*c*_	=70 *μ*s	coherence time of the qubit
*T*_1_	=70 *μ*s	qubit *T*_1_ coherence time
*T*_2_	=70 *μ*s	qubit *T*_2_ coherence time
Γ_gas_	=2.7 × 10^−8^ Hz	resonator amplitude damping rate due to background gas collisions
Γ_eddy_	=8.1 × 10^−19^ Hz	resonator amplitude damping rate due to induced eddy current losses
Γ_rad_	=3.3 × 10^−22^ Hz	resonator amplitude damping rate due to magnetic dipole radiation
