# Supplementary material for: Macroscopic superpositions and gravimetry with quantum magnetomechanics
Source: Sci Rep. 2016 Nov 21;6:37495. doi: 10.1038/srep37495 (PMC5116620; doi:10.1038/srep37495)
Supplement: Supplementary Information [file srep37495-s1.pdf]

Supplementary Materials to the Manuscript: Macroscopic superpositions and gravimetry with quantum magnetomechanics

Mattias T. Johnsson, Gavin K. Brennen, and Jason Twamley

Centre for Engineered Quantum Systems, Department of Physics and Astronomy, Macquarie University, Sydney, NSW 2109, Australia

Appendix A: Vertical and transverse trapping of the resonator

In cylindrical coordinates (ρ, ϕ, z), with the origin at the centre of the sphere, the vector potential for the magnetized sphere described in the main paper is

$$\mathbf{A}(\mathbf{r}) = \frac{\mu_0 \mathcal{M} V \rho}{4\pi(\rho^2 + z^2)^{3/2}} \hat{\phi}, \tag{A1}$$

where V is the sphere volume. Since $\mathbf{B} = \nabla \times \mathbf{A}$ in cylindrical coordinates we have

$$\mathbf{B}(\mathbf{r}) = \frac{\mu_0 \mathcal{M} V}{4\pi(\rho^2 + z^2)^{5/2}} \begin{bmatrix} 3z\rho \\ 0 \\ 2z^2 - \rho^2 \end{bmatrix}. \tag{A2}$$

To calculate the vertical trapping frequency ω, we require the force exerted on the resonator ring as a function of displacement from the equilibrium point. This force arises due to the fact that when the ring moves, the magnetic flux threading through it will change. As flux lines can't pass through the superconducting ring, however, a current arises in the ring to restore the flux, and this current gives rise to a Lorentz force.

Since the resonator ring is horizontal, the flux through it is given by the line integral

$$\Phi = \oint \mathbf{A}(\mathbf{r}) \cdot d\mathbf{r} = \frac{\mu_0 \mathcal{M} V R_r^2}{2(R_r^2 + z^2)^{3/2}}. \tag{A3}$$

Changes in current in the resonator as it moves vertically are related to changes in flux via

$$\frac{dI_r}{dz} = -\frac{1}{L_r}\frac{d\Phi}{dz}, \tag{A4}$$

where L_r is the self inductance of the ring and is given by $L_r = \mu_0 R_r(\ln[8R_r/a] - 2)$.

Taking the equilibrium vertical position point $z = z_{\rm eq}$, then for small displacements the current in the resonator is $I_r = (z - z_{\rm eq}) \cdot dI_r/dz|_{z=z_{\rm eq}}$ where, using (A4),

$$\left.\frac{dI_r}{dz}\right|_{z=z_{\rm eq}} = \frac{3\mu_0 \mathcal{M} V R_r^2 z_{\rm eq}}{2L(R_r^2 + z_{\rm eq}^2)^{5/2}}. \tag{A5}$$

The Lorentz force from the current, magnitude I_r, flowing through a small element $\mathbf{dl}$ of the wire is given by $\mathrm{d}\mathbf{F} = (I_r\, \mathrm{d}\mathbf{l}) \times \mathbf{B}$. Assuming the resonator is circular, sitting horizontally, and is co-axial with the $\hat{z}-$axis, the line element $\mathrm{d}\mathbf{l}$ will always be perpendicular to $\mathbf{B} = B_{\text{radial}}\hat{\rho} + B_{\text{axial}}\hat{z}$. Hence the vertical force on the resonator for small vertical displacements from equilibrium $z - z_{\text{eq}}$, is

$$F_z = -I_r \int_0^{2\pi} B_{\text{radial}} R_r d\phi = -\frac{9\mu_0^2 \mathcal{M}^2 V^2 R_r^4 z_{\text{eq}}^2 (z - z_{\text{eq}})}{4L_r (R_r^2 + z_{\text{eq}}^2)^5}. \tag{A6}$$

Finally, the equation of motion in the z direction is

$$\frac{d^2 z}{dt^2} = \frac{F_z}{m} = -\omega^2 (z - z_{\text{eq}}) \tag{A7}$$

for small displacements, providing an harmonic restoring force. Comparing (A6) and (A7) we find the vertical oscillation frequency

$$\omega = \frac{3\mu_0 \mathcal{M} V R_r^2 z_{\text{eq}}}{2\sqrt{m L_r (R_r^2 + z_{\text{eq}}^2)^5}}\,. \tag{A8}$$

We also need to consider transverse trapping and oscillations firstly to establish that the resonator is indeed trapped in all three directions, and secondly to determine if there is any coupling between the vertical and horizontal motions. If this coupling exists then by cooling the vertical motion one cools the entire motion of the resonator, but such couplings can also lead to unwanted energy leakage from the coherent vertical dynamics to the transverse modes, leading to decoherence of our vertical superposition states.

When considering the horizontal movement of the resonator we break the cylindrical symmetry, meaning it is easier to work in Cartesian coordinates. In this coordinate system the magnetic vector potential is given by

$$\mathbf{A}(\mathbf{r}) = \frac{\mu_0 \mathcal{M} V}{4\pi (x^2 + y^2 + z^2)^{3/2}} \begin{bmatrix} -y \\ x \\ 0 \end{bmatrix}. \tag{A9}$$

Due to the coordinate system, rather than a circular resonator, we consider a square resonator of width $2w$, and wire radius a, and assume it is displaced sideways along the $x-$axis a small amount δx.

We can calculate the flux through the resonator at a displaced position δx via Eq. (A3), and

expand the result in a Taylor series in δx. To third order we get

$$\Phi(\delta x) = \frac{2\mu_0 w^2 \mathcal{M}}{\pi(w^2+z^2)\sqrt{2w^2+z^2}} + \frac{\mu_0 w^2 \mathcal{M} V (5w^6 - 11w^4z^2 - 18w^2z^4 - 6z^6)}{\pi(w^2+z^2)^3(2w^2+z^2)^{5/2}}\delta x^2 + O[\delta x^4]\,. \tag{A10}$$

The zeroth-order term is a constant for motion along the $x-$direction and can be ignored. Using (A4) modified for $x-$directional motion we obtain the dependence of the induced current on δx,

$$I(\delta x) = -\frac{w\mathcal{M}V(5w^6 - 11w^4z^2 - 18w^2z^4 - 6z^6)}{4(w^2+z^2)^3(2w^2+z^2)^{5/2}(\log[2w/a] - 0.774)}\delta x^2\,, \tag{A11}$$

where we have used the fact that self-inductance of a square loop is $L = 2\mu_0 w(\log[w/a] - 0.774)/\pi$. Using the Lorentz force law as in the previous section, we can integrate the loop current in the presence of the magnetic field and obtain the resulting force. Renaming the small displacements $\delta x \to x$ and similarly for y, z from the equilibrium point $(0, 0, z_{\mathrm{eq}})$, we find that to lowest order the $x-$component of this force is $F_x = -\beta x^3$ with $\beta > 0$, and at equilibrium the resonator is transversely trapped in a pure anharmonic potential. As these forces come from a conservative potential we can integrate along paths to obtain the leading terms for the potential of the system

$$V = \frac{1}{2}m\omega^2 z^2 + \frac{1}{3}\gamma(x^2+y^2)z + \frac{1}{4}\beta(x^4+y^4)\,, \tag{A12}$$

which describes a type of cross-mode coupling. For parameters described in Table I we find $(m\omega^2/2, \gamma, \beta) = (1.73\times10^{-2}\ \mathrm{J},\ 1.98\times10^{3}\ \mathrm{Jm^{-1}},\ 2.65\times10^{8}\ \mathrm{Jm^{-2}})$.

Appendix B: Cooling to the ground state

In order to put our resonator into a cat state and use it as a gravimeter, it is necessary to ensure that we can begin with it in the motional ground state. This in turn requires that we have mechanism to cool it from its initial non-equilibrium state to the ground state by removing energy.

Details of the cooling scheme we use can be found in Refs. [1, 2], which we briefly summarize here. We cool by coupling a two level system (the qubit) to the resonator, with the qubit coupled to a bosonic thermal bath. The Hamiltonian for the coupled system is

$$\hat{H} = -\frac{\hbar\delta}{2}\hat{\sigma}^z + \frac{\hbar\Omega}{2}\hat{\sigma}^x + \hbar\omega\hat{a}^\dagger\hat{a} + \frac{\hbar\lambda}{2}(\hat{a}+\hat{a}^\dagger)\hat{\sigma}^z \tag{B1}$$

where Ω is the Rabi frequency with which we drive the qubit, δ is the detuning of the driving field from resonance with the qubit frequency splitting ω_q, $\hat{a}$ is the annihilation operator for the resonator oscillation modes and the $\hat{\sigma}^{x,z}$ are the standard spin-1/2 Pauli operators.

The open systems dynamics of the qubit-resonator system is described in Sec. E and is characterised by Γ and $\Gamma_\perp$, the amplitude damping rates of the resonator and qubit respectively. The initial state of the resonator is modelled as a coherent state with amplitude $\alpha = \sqrt{N_{\rm th}}$ where the initial occupation number is $N_{\rm th} = (e^{\hbar\omega/k_B T_r} - 1)^{-1}$ with T_r an effective bath temperature for the environment of the resonator. In the limit where $\lambda \ll \Gamma_\perp, \omega$, the final phonon occupation number for the resonator, n_f, is given by

$$n_f = N_{\rm th}[\zeta + (1-\zeta)/(1+\zeta \exp[I_1/(N_{\rm th}\zeta(\lambda/\omega)^2)])]. \tag{B2}$$

Here $\zeta = \Gamma/\Gamma_c(0)$ and the renormalized cooling rate is $\Gamma_c(\alpha) = i\lambda(\vec{S}^z_1/\alpha - \vec{S}^z_{-1}/\alpha^*)$, with $I_1 = 2\int_0^\infty d\alpha\, \alpha\tilde{\Gamma}_c(\alpha\omega/\lambda)$ and $\tilde{\Gamma}_c = \Gamma_c(\alpha)/\Gamma_c(0)$. The qubit polarization Fourier components, $\vec{S}^z_1$ and $\vec{S}^z_{-1}$, are given by the solutions to the Bloch equations for the qubit. In the Lamb-Dicke regime ($\lambda\sqrt{N_{\rm th}+1/2} \ll \Gamma_\perp, \omega$) one can obtain an effective master equation for the resonator after tracing out the qubit. This gives a new effective resonator damping rate $\Gamma_{\rm cool} = \Gamma_c + \Gamma$ with $\Gamma_c = S(\omega) - S(-\omega)$ where $S(\nu)$ denotes the qubit fluctuation spectrum and is given by

$$S(\nu) = \frac{\lambda^2}{2}{\rm Re}\int_0^\infty e^{i\nu t}dt[\langle\hat{\sigma}^z(t)\hat{\sigma}^z(0)\rangle_0 - \langle\hat{\sigma}^z(0)\rangle_0^2], \tag{B3}$$

where $\langle\cdot\rangle_0$ denotes the steady state expectation. The resulting steady state phonon occupation of the resonator in the Lamb-Dicke regime is [2]

$$n_{LD} = \Gamma N_{\rm th}/\Gamma_c + N_0, \tag{B4}$$

where $N_0 = S(-\omega)/\Gamma_c$.

In Figure 1 we plot the performance of this cooling scheme for our system, showing both the full cooling solution and a simplified cooling solution that makes the assumption that we are always in the Lamb-Dicke regime, i.e. Eq. (B4) holds for all initial resonator temperatures. The plot shows that even with initial phonon occupation numbers as high as $\sim 10^9$ we can cool the resonator to the ground state, with an average final occupation number of 0.16.

The timescale governing the cooling is given by the effective resonator cooling rate $\Gamma_{\rm cool}$. For our system, using parameters given in the caption of Figure 1, we obtain $\Gamma_{\rm cool} = 27$ kHz. We note this cooling rate scales as λ^2, and we have chosen a very conservative coupling rate of $\lambda = 10$ kHz.

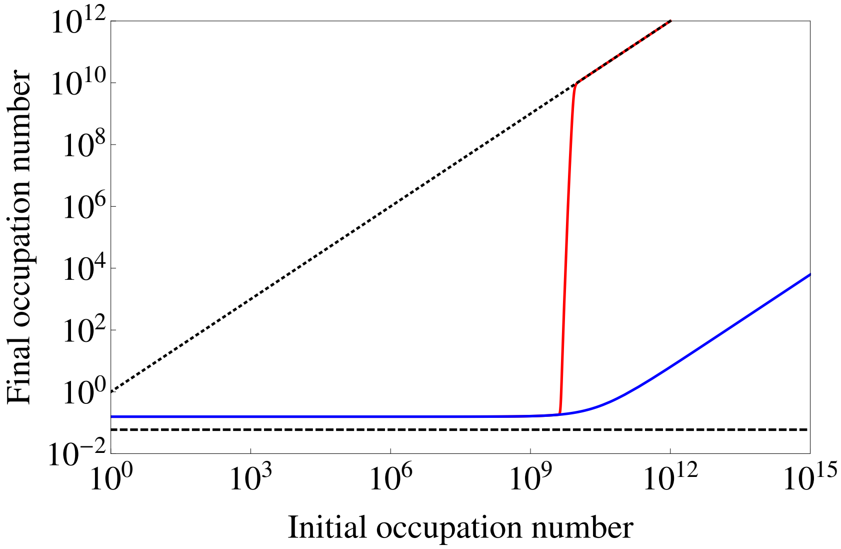

FIG. 1. Cooling performance of our system. Horizontal dashed black line shows the phonon occupation of the qubit in a temperature bath of $T_q = 100\,\mathrm{mK}$, solid blue line shows the final phonon occupation number of the resonator after cooling assuming the Lamb-Dicke regime is valid, and the solid red line shows the exact cooling solution (note blue and red lines overlap for low initial occupation numbers). The diagonal dotted black line shows the line where initial and final occupation numbers are the same; any part of the blue or red curves below this represents cooling. The plot shows that even with initial phonon occupation numbers as high as $\sim 10^9$ we can cool the resonator to the ground state, with an average final occupation number of 0.16. We have assumed the standard resonator parameters given in Table I, and take $\Omega = \omega/2$, $\delta = -\sqrt{\omega^2 - \Omega^2}$, $\lambda/2\pi = 10^4\,\mathrm{Hz}$, decoherence times $T_1 = T_2 = 70\,\mu\mathrm{s}$, $\omega_q/2\pi = 6\,\mathrm{GHz}$, and $\Gamma = 2.70 \times 10^{-8}\,\mathrm{Hz}$. See Table I for the definitions of system parameters.

As our system is capable of coupling strengths of up to $\lambda \sim 1\,\mathrm{GHz}$, the cooling can be made much faster if required.

Appendix C: Ancillary parameter determination

While our measurement protocol and phase estimation scheme gives us a phase, this phase must still be converted to a value for g via

$$\phi = (2mgl - \hbar\omega_q)\,\frac{2\pi}{\hbar\omega}. \tag{C1}$$

Clearly, in order to obtain a precise estimate of g, we must know the parameters m, ω, ω_q and λ to the same level of precision. These quantities can be measured offline with any additional

resources, and will not affect the time taken for the phase estimation protocol.

One way to obtain information on these parameters is to observe the effect on the evolution of the qubit. If the qubit is not driven, i.e. $\Omega = 0$, then the equations of motion associated with the full coupled Hamiltonian

$$\hat{H} = \frac{\hbar\omega_q}{2}\hat{\sigma}^z + \hbar\omega\hat{a}^\dagger\hat{a} + \frac{\hbar\lambda}{2}(\hat{a} + \hat{a}^\dagger)\hat{\sigma}^z + mg\hat{z} \tag{C2}$$

are

$$\begin{aligned} \frac{d\hat{a}}{dt} &= -i\omega\,\hat{a} - \frac{i\lambda}{2}\hat{\sigma}^z - i\sqrt{\frac{mg^2}{2\hbar\omega}} \\ \frac{d\hat{\sigma}_x}{dt} &= 2\omega_q\,\hat{\sigma}^y - \lambda(\hat{a} + \hat{a}^\dagger)\hat{\sigma}^y \\ \frac{d\hat{\sigma}_y}{dt} &= -2\omega_q\,\hat{\sigma}^x + \lambda(\hat{a} + \hat{a}^\dagger)\hat{\sigma}^x \\ \frac{d\hat{\sigma}_z}{dt} &= 0. \end{aligned} \tag{C3}$$

If we assume the resonator starts in the ground state then we have $\langle\hat{a}(0) + \hat{a}^\dagger(0)\rangle = 0$. Denoting $\sigma^i = \langle\hat{\sigma}^i\rangle$ and $a = \langle\hat{a}\rangle$, Eqs. (C3) have the solution

$$\begin{aligned} a(t) + a^*(t) &= \frac{\lambda\sigma^z(0)}{\omega}(\cos[\omega t] - 1) \\ \sigma^x(t) &= \sigma^x(0)\cos\xi + \sigma^y(0)\sin\xi \\ \sigma^y(t) &= \sigma^y(0)\cos\xi - \sigma^x(0)\sin\xi \\ \sigma^z(t) &= \sigma^z(0)\,, \end{aligned} \tag{C4}$$

where

$$\xi = 2\omega_q t + \frac{\sigma^z(0)\lambda^2}{\omega} - \frac{\sigma^z(0)\lambda^2\sin[\omega t]}{\omega^2}. \tag{C5}$$

These solutions have intricate time-dependent structure, meaning an arbitrary number of independent datapoints can be obtained by measuring, say, $\hat{\sigma}^x$ on the qubit. Provided the qubit preparation and measurement process has only statistical errors and not systematic ones, arbitrarily precise values of ω_q, ω and λ can be obtained by fitting a suitably large number of measurement results against the theoretically expected profile.

In order to measure the mass of the resonator, techniques such as those described by Schilling are likely to perform well [3, 4]. These schemes utilize electro-optical measurement of oscillation period of a levitated superconducting oscillator, exactly the same situation as described by our scheme.

Of course, if other simpler or more precise methods are available that can provide values for any of these parameters, they can be used in the calibration process and reduce the number of parameters that need to be fitted.

Appendix D: Decoherence

1. Quality Factor

In the subsequent discussions we make use of the quality factor Q of the mechanical oscillations of our resonator. One usual definition of Q is given by

$$Q = \frac{\hbar\omega^2}{P}, \tag{D1}$$

where P is the power loss, ω is the oscillation frequency and $\hbar\omega$ is the energy of the system. In our protocol, however, the resonator is not in the motional ground state — the resonator is oscillating back and forth with a large amplitude (several nanometers). As we begin the interferometry protocol (or *slosh*), with the resonator high up on a potential hill, we have $V(l) = \frac{1}{2}m\omega^2 l^2$ where $l = \lambda z_0/\omega$ is the displacement from equilibrium, and $z_0 = \sqrt{\hbar/2m\omega}$ is the harmonic oscillator ground state extent. This means for our system we have $V = \hbar\lambda^2/4\omega$. Associating this potential energy with the energy in (D1) we obtain

$$Q \approx \frac{\hbar\lambda^2}{4P}. \tag{D2}$$

To compute an associated decoherence rate we use

$$\Gamma = \omega/2\pi Q. \tag{D3}$$

We also note that for all the calculations in this section we use the system parameters described in Table I.

2. Qubit dephasing

The effect of qubit decoherence on the evolution of the joint qubit-resonator system is solved for in Supp Material E, The main result is that the off diagonal elements of the qubit density matrix, which carry the gravitationally induced phase accumulation, will experience exponential decay by a factor $e^{-\tau/T_2}$ where T_2 is the qubit dephasing time. Dephasing rates vary greatly with

the superconducting circuit architecture. Recent experiments with superconducting flux qubits in 3D microwave cavities have reported decoherence times of $T_2^{\rm echo} > 19\,\mu$s [5], while decoherence times of $T_2^{\rm echo} > 100\,\mu$s have been reported for transmon qubits in 3D cavities [6].

3. Decoherence due to eddy currents in the magnet

As the resonator oscillates, carries currents, and is in close proximity to the magnetised sphere, it will inductively induce eddy currents in the sphere, which will result in power loss as the magnetic material has electrical resistance. In order to estimate this effect we consider infinitesimal horizontal loops of radius R' inside the sphere and placed at a distance h from the bottom of the sphere. The electromotive force induced in each of such loops due to the resonator motion is given by $|\epsilon| = M_{l,s}(R', h)\, dI_r/dt$, where $M_{l,s}$ is the mutual inductance between the horizontal loop of the resonator and the horizontal infinitesimal loop of the sphere.This gives an upper bound on the power loss as

$$\begin{aligned} P &\leq \int \frac{\epsilon^2}{\rho 2\pi} dR'\, dh \\ &= \left(\frac{\mu_0}{4\pi}\right)^2 \frac{2\pi^3 R_r^4 I_r^2 \omega^2}{\rho} \int_{h=0}^{2R_s} dh \int_0^{\sqrt{R_s^2-(R_s-h)^2}} dR' \frac{R'^3}{(r_0+h)^6} \\ &= \left(\frac{\mu_0}{4\pi}\right)^2 \frac{2\pi^3 R_r^4 I_r^2 \omega^2}{\rho} \frac{4R_s^5}{15 r_0^3 (r_0+2R_s)^3}, \end{aligned} \tag{D4}$$

where r_0 is the minimum distance from the bottom of the sphere to the centre of the resonator, and ρ is the resistivity of the magnetic material. Our sphere is composed of YIG, which has $\rho = 10^{12}\,\Omega$m; we take the I_r to be the largest current reached in the resonator (occurring at full displacement), i.e. $I_{r\max} \sim 48\,\mu$A. This gives the power loss due to eddy currents in the YIG sphere as $P = 6.2 \times 10^{-38}$ W, which via (D2) corresponds to a quality factor of $Q = \hbar\omega^2/P = 3.1 \times 10^{22}$ and a decoherence rate of $\Gamma_{\rm eddy} = 8.1 \times 10^{-19}$ s^{-1}.

4. Dipole radiation

An oscillating loop carrying current will emit electromagnetic radiation, dissipating energy from our system. We treat our resonator loop as a dipole, with a current given by $I = I_{r\max} e^{i\omega t}$. The power loss of an oscillating dipole due to radiation is given by $P = R_{\rm rad} I^2/2$, where $R_{\rm rad} = \frac{\pi}{6}\left(\frac{R_r\omega}{c}\right)^4 Z$, where $Z = 377\,\Omega$ is the impedance of the vacuum. Using the parameters in Table I we

obtain a power loss of $P = 2.5 \times 10^{-41}$ W, corresponding to $Q = 7.5 \times 10^{25}$, and an associated decoherence rate of $\Gamma_{\rm rad} = 3.3 \times 10^{-22}\,{\rm s}^{-1}$.

5. Background gas collisions

In the limit where the mean free path of the gas molecules is sufficiently large, the damping rate is given by [7]

$$\Gamma = 2\rho_{\rm gas} A\, u_{\rm av}/m_g, \tag{D5}$$

where $\rho_{\rm gas}$ is the density of the gas, $A = 2\pi R_r 2a$ is the cross-sectional area of the resonator interacting with the gas, m_g is the mass of a gas molecule, and $u_{\rm av} = \sqrt{2k_B T/m_g}$ is the average velocity of a gas molecule. In order to be in this limit, the system must have a Knudsen number Kn > 10 [8]. Using the parameters in Table I, our system has Kn $\sim 10^9$, far into regime where Eq. (D5) is valid. Taking $m_g = 3.98 \times 10^{-26}$ kg (nitrogen molecule), $T = 0.1$ K, a resonator ring radius of $5\,\mu$m, a resonator wire radius of $1.0\,\mu$m, and area of $A = 6.28\times10^{-11}$ m^2, and a pressure of 10^{-9} Pa, Eq. (D5) yields $\Gamma_{\rm gas} = 2.7 \times 10^{-8}$ Hz and an associated $Q = 9.2 \times 10^{11}$.

6. Coupling to torsional modes

Coupling of the vertical (z) centre of mass ($z-$COM) oscillation mode to other bending/twisting/torsional modes of the resonator also allows energy to leak from the $z-$COM phonon mode. Of these alternative motional modes one can consider, the lowest frequency mode is the torsional mode which has frequencies

$$\nu = \frac{1}{2\pi}\sqrt{\frac{EA}{2\mu R_r^2}}\sqrt{1+n^2}\,, \tag{D6}$$

where $n > 0$ is the integer valued mode number, A is the cross sectional area of the wire, $\mu = \rho\pi a^2$ is the mass per unit circumference, and E is the Young's modulus of the wire. We take $E = 16 \times 10^9$ Pa, $\rho = 11340$ kg/m^3 giving $\mu = 3.56 \times 10^{-8}$ kg/m. This gives the lowest frequency mode as $\nu = 1.89 \times 10^8$ rad/s, which is ~ 1200 times larger than ω, indicating cross-coupling to other modes is negligible.

Appendix E: Open System Dynamics

The open systems dynamics of the joint qubit-resonator system is given by the master equation

$$\dot{\hat{\rho}}(t) = \hat{\mathcal{L}}(\rho(t)) \tag{E1}$$

with the Louivillian

$$\hat{\mathcal{L}} = -\frac{i}{\hbar}[\hat{H}, \rho(t)] + \hat{\mathcal{L}}_r + \hat{\mathcal{L}}_q.$$

Free evolution is governed by the Hamiltonian

$$\hat{H} = \frac{\hbar\omega_q}{2}\hat{\sigma}_z + \frac{\hat{p}^2}{2m} + \frac{1}{2}m\omega^2\left(\hat{z} + l\hat{\sigma}_z\right)^2 - mgl\hat{\sigma}_z,$$

and amplitude damping of the resonator and amplitude and phase damping of the qubit are described by:

$$\begin{aligned} \hat{\mathcal{L}}_r &= \frac{\Gamma}{2}\hat{D}[\hat{a}] \\ \hat{\mathcal{L}}_q &= \frac{\Gamma_\perp}{2}(N_q + 1)\hat{D}[\hat{\sigma}^-] + \frac{\Gamma_\perp}{2}N_q\hat{D}[\hat{\sigma}^+] + \frac{\Gamma_\parallel}{4}\hat{D}[\hat{\sigma}^z]\ , \end{aligned} \tag{E2}$$

with the map $\hat{D}$ defined as

$$\hat{D}[\hat{O}](\hat{\rho}) \equiv 2\hat{O}\hat{\rho}\hat{O}^\dagger - \{\hat{O}^\dagger\hat{O}, \hat{\rho}\}.$$

The equilibrium phonon occupation of the qubit environment is $N_q = (e^{-\hbar\omega_q/k_B T_q} - 1)^{-1}$ where T_q is the qubit phonon bath temperature. The decay rates are related to the usual decoherence times according to $T_1^{-1} \equiv \Gamma_\perp(2N_q + 1)$ and $T_2^{-1} \equiv T_1^{-1}/2 + \Gamma_\parallel$. We treat the environment of the resonator as zero temperature meaning the resonator only loses energy to the environment. This is justified as it is not clamped to any material and we assume the surrounding cavity is in the electromagnetic vacuum state. Any temperature dependence of damping due to background gas collisions can be encorpeated into the value of damping rate Γ_{gas} as described in Sec. D 5.

At each measurement run, the joint state of the qubit and resonator is prepared in the initial state

$$\hat{\rho}(0) = \frac{1}{2}\begin{pmatrix} 1 & 1 \\ 1 & 1 \end{pmatrix}_q \otimes |0\rangle_r\langle 0|\ , \tag{E3}$$

where $|0\rangle_r$ is the motional ground state of the resonator. At this point we can make some simplifications. We are interested in obtaining a worst case scaling for the decoherence of our protocol which would occur when the size of the initial Schrödinger cat state is largest, i.e. $l = l_{\max}$. The

time evolution is only over one period of oscillation $\tau = 2\pi/\omega$ of the resonator and we assume that $\Gamma_\perp, \Gamma_\| < \omega$ and $\Gamma \ll \omega$. It is convenient to divide the Louivillian into two parts: $\hat{\mathcal{L}} = \hat{\mathcal{L}}_1 + \hat{\mathcal{L}}_2$:

$$\hat{\mathcal{L}}_1 = -\frac{i}{\hbar}[\hat{H}, \cdot] + \hat{\mathcal{L}}_r + \frac{\Gamma_\|}{4} D[\hat{\sigma}^z]$$

and

$$\hat{\mathcal{L}}_2 = \frac{\Gamma_\perp}{2}(N_q + 1) D[\hat{\sigma}^-] + \frac{\Gamma_\perp}{2} N_q D[\hat{\sigma}^+]$$

During evolution generated by $\hat{\mathcal{L}}_1$, the operator $\hat{\sigma}^z$ is a conserved quantity and we can solve for the joint evolution of the qubit and resonator exactly. Evolution generated by $\hat{\mathcal{L}}_2$ describes amplitude damping of the qubit. We approximate the evolution of the system over one resonator oscillation period τ as the composition of maps:

$$\mathcal{E}^{(\text{evA})}(\hat{\rho}(0)) \equiv e^{\hat{\mathcal{L}}\tau}(\hat{\rho}(0)) \approx e^{\hat{\mathcal{L}}_2\tau} \circ e^{\hat{\mathcal{L}}_1\tau}(\hat{\rho}(0)).$$

We first consider evolution by $\hat{\mathcal{L}}_1$. The qubit dephasing simply introduces decay of off diagonal qubit states. Damping maps coherent states to coherent states and since we begin in a superposition of coherent states, at any time t we can write the joint state in the interaction picture $\hat{\rho}_I(t) = e^{i\hat{H}t}\hat{\rho}e^{-i\hat{H}t}$ as

$$\hat{\rho}_I(t) = \sum_{M,M'=-1}^{1} c_{M,M'} e^{-\frac{\gamma_Q}{2}|M-M'|t} |M\rangle\langle M'| \otimes \hat{A}_I^{M,M'}(t),$$

where the eigenbasis of $\hat{\sigma}^z$ is $|M = \pm 1\rangle$ and

$$\hat{A}_I^{M,M'}(t) = |\alpha_I^M(t)\rangle\langle\beta_I^{M'}(t)| \, .$$

To derive the evolution during decay we use the characteristic function

$$X(t) = \text{Tr}_R[A_I^{M,M'}(t) e^{\Lambda \hat{a}_I^\dagger} e^{-\Lambda^* \hat{a}_I}] \, ,$$

where the trace is taken over the resonator's motional degree of freedom such that

$$\dot{X}(t) = \text{Tr}_R[\dot{\hat{A}}_I^{M,M'}(t) e^{\Lambda \hat{a}_I^\dagger} e^{-\Lambda^* \hat{a}_I}] \tag{E4}$$

$$= \Gamma \text{Tr}_F\Big[(\hat{a}_I \hat{A}_I^{M,M'}(t) \hat{a}_I^\dagger - \frac{1}{2}\hat{a}_I^\dagger \hat{a}_I \hat{A}_I^{M,M'}(t) - \tag{E5}$$

$$\frac{1}{2}\hat{A}_I^{M,M'}(t) \hat{a}_I^\dagger \hat{a}_I) e^{\Lambda \hat{a}_I^\dagger} e^{-\Lambda^* \hat{a}_I}\Big]. \tag{E6}$$

Using the relations

$$e^{-\Lambda^* \hat{a}} \hat{a}^\dagger = (\hat{a}^\dagger - \Lambda^*) e^{-\Lambda^* \hat{a}}, \quad \hat{a} e^{\Lambda \hat{a}^\dagger} = e^{\Lambda \hat{a}^\dagger}(\hat{a} + \Lambda) \, ,$$

we obtain

$$\dot{X} = -\frac{\Gamma}{2}\Big(\Lambda^* \frac{\partial \hat{X}}{\partial \Lambda^*} + \Lambda \frac{\partial \hat{X}}{\partial \Lambda}\Big)$$
$$= -\frac{\Gamma}{2}\Big(\beta_I^{M'*}(t)\Lambda - \alpha_I^M(t)\Lambda^*\Big)X \ . \tag{E7}$$

To solve for the dynamics, we make the ansatz:

$$X(t) = C(t)e^{-\lambda^* \alpha_I^M(t)} e^{\lambda \beta_I^{M'*}(t)} \ . \tag{E8}$$

From the reflection symmetry of the state dependent traps, the magnitudes of the coherent states correlated with the qubit states are equal at all time so we can write $\beta_I^{M'}(t) = \alpha_I^{M'}(t)$. Evaluating the time derivative of $X(t)$ and setting this equal to Eq. (E7) we can solve for the dynamics. The diagonal terms evolve as

$$e^{\hat{\mathcal{L}}_r t}[|\alpha_I^M(0)\rangle\langle\alpha_I^M(0)|] = |\alpha_I^M(t)\rangle\langle\alpha_I^M(t)| \ .$$

The off-diagonal terms evolve as

$$\begin{aligned} e^{\hat{\mathcal{L}}_r t}[|\alpha_I^M(0)\rangle\langle\alpha_I^{-M}(0)|] &= |\alpha_I^M(t)\rangle\langle\alpha_I^{-M}(t)| \ \langle\alpha_I^{M'}(0)|\alpha_I^M(0)\rangle^{1-e^{-\Gamma t}} \\ &= |\alpha_I^M(t)\rangle\langle\alpha_I^{-M}(t)| \exp[-\tfrac{1}{2}(|\alpha_I^M(0)|^2 \\ &\quad +|\alpha_I^{-M}(0)|^2 - 2\alpha_I^M(0)\alpha_I^{M'*}(0)]^{1-e^{-\Gamma t}} \\ &= |\alpha_I^M(t)\rangle\langle\alpha_I^{-M}(t)| \exp[-2\lambda^2/\omega^2]^{1-e^{-\Gamma t}} \ . \end{aligned} \tag{E9}$$

Transforming back to the Schrödinger picture, the state written explictly in the qubit basis is:

$$\hat{\rho}(t) = \frac{1}{2}\begin{pmatrix} |\alpha^1(t)\rangle\langle\alpha^1(t)| & e^{ict}e^{\kappa(t)}|\alpha^1(t)\rangle\langle\alpha^{-1}(t)| \\ e^{-ict}e^{\kappa(t)}|\alpha^{-1}(t)\rangle\langle\alpha^1(t)| & |\alpha^{-1}(t)\rangle\langle\alpha^{-1}(t)| \end{pmatrix}_Q , \tag{E10}$$

where

$$|\alpha^M(t)\rangle = |(1 + e^{-\Gamma t/2}e^{i\omega t})\lambda M/2\omega\rangle \ ,$$

the coherently evolved phase is

$$c = 2mgl/\hbar - \omega_q \ ,$$

and

$$e^{\kappa(t)} = \exp[-2\lambda^2/\omega^2]^{1-e^{-\Gamma t}} e^{-\Gamma_\parallel t} \ .$$

We seek a form for the joint state after one oscillation period $\tau = 2\pi/\omega$. Since $2\pi\Gamma/\omega = Q^{-1} \ll 1$, we can approximate $\alpha^M(2\pi/\omega) \approx \alpha^M(0)$ and $1 - e^{-\Gamma 2\pi/\omega} \approx \Gamma\tau$, so that

$$e^{\hat{\mathcal{L}}_1 \tau}(\hat{\rho}(0)) = \frac{1}{2}\begin{pmatrix} 1 & e^{i\phi}e^{-(\Gamma_\parallel+\gamma)\tau} \\ e^{-i\phi}e^{-(\Gamma_\parallel+\gamma)2\pi/\omega} & 1 \end{pmatrix}_q \otimes |0\rangle_r\langle 0| \ , \tag{E11}$$

where the coherent phase is

$$\phi = \frac{2\pi}{\omega}(2mgl/\hbar - \omega_q) , \tag{E12}$$

and the decoherence is governed by the factor

$$\gamma = \frac{2\Gamma l^2}{z_0^2} . \tag{E13}$$

As expected, the dephasing grows with the square of the cat state separation.

Evolution according to $\hat{\mathcal{L}}_2$ is a map that acts only on the qubit and can be solved for explicitly. The full evolution over one oscillation period returns the joint system to a product state of the resonator in the vacuum motional state and the qubit in a mixed state:

$$\mathcal{E}^{(\mathrm{evA})}(\hat{\rho}(0)) \approx \begin{pmatrix} T_1\Gamma_\perp(1+N_q-\frac{e^{-\tau/T_1}}{2}) & \frac{1}{2}e^{i\phi}e^{-\gamma\tau}e^{-\tau/T_2} \\ \frac{1}{2}e^{-i\phi}e^{-\gamma\tau}e^{-\tau/T_2} & T_1\Gamma_\perp(N_q+\frac{e^{-\tau/T_1}}{2}) \end{pmatrix}_q \otimes|0\rangle_r\langle 0| . \tag{E14}$$

This is the expression used in the overall measurement fidelity in Eq. (33) in the main text where the gravitationally induced phase ϕ is obtainable by measuring qubit coherences.

[1] Cirio, M., Brennen, G. K. & Twamley, J. Quantum Magnetomechanics: Ultrahigh-Q-Levitated Mechanical Oscillators. *Phys Rev Lett* **109**, 147206 (2012).

[2] Rabl, P. Cooling of mechanical motion with a two-level system: The high-temperature regime. *Phys Rev B* **82**, 165320 (2010).

[3] Schilling, O. F. The precise determination of mass through the oscillations of a very high-q electromechanical system. *Journal of Electromagnetic Analysis and Applications* **5**, 91 (2013).

[4] Schilling, O. F. The precise determination of mass through the oscillations of a very high-q superconductor oscillating system. *arXiv:1309.0699* (2013).

[5] Stern, M. *et al.* Flux qubits with long coherence times for hybrid quantum circuits. *Phys Rev Lett* **113**, 123601 (2013).

[6] Geerlings, K. L. *Improving Coherence of Superconducting Qubits and Resonators*. Ph.D. thesis, Yale University (2013).

[7] Guccione, G. *et al.* Scattering-Free Optical Levitation of a Cavity Mirror. *Phys Rev Lett* **111**, 183001 (2013).

[8] Bhiladvala, R. B. & Wang, Z. J. Effect of fluids on the Q factor and resonance frequency of oscillating micrometer and nanometer scale beams. *Phys Rev E* **69**, 036307 (2004).

TABLE I. System parameters and the values used in the main text for precision gravimetry.

Symbol	Value	Definition
Φ_0	$= 2.07 \times 10^{-15}$ Wb	flux quantum
g	$= 9.81\,\mathrm{m\,s^{-2}}$	acceleration due to gravity
m	$= 1.12 \times 10^{-12}$ kg	resonator mass (Pb)
$\omega/2\pi$	$= 24.8$ kHz	resonator frequency
z_0	$= 1.74 \times 10^{-14}$ m	ground state rms width of resonator
R_q	$= 5\,\mu$m	radius of qubit loop
R_r	$= 5\,\mu$m	radius of resonator ring
R_{sphere}	$= 10\,\mu$m	radius of magnetized sphere
a	$= 1.0\,\mu$m	radius of resonator wire
d	$= 2.0\,\mu$m	distance between resonator centre of mass and qubit
r_0	$= 1\,\mu$m	minimum distance from sphere surface to centre of mass of resonator
z_{eq}	$= 11\,\mu$m	equilibrium position of resonator
V	$= 4.19 \times 10^{-15}\,\mathrm{m^3}$	volume of magnetised sphere
$\mathcal{M}$	$= 8.76 \times 10^{2}\,\mathrm{A\,m^{-1}}$	magnetisation of YIG sphere
ρ	$= 10^{12}\,\Omega$m	resistivity of magnetised sphere made of YIG
l_{max}	$= 9.5 \times 10^{-10}$ m	largest size of Schrödinger cat
$\lambda_{\text{max}}/2\pi$	$= 1.35$ GHz	maximum qubit-resonator coupling
$\lambda_0/2\pi$	$= 0.63$ Hz	minimum qubit-resonator coupling
L_r	$= 2.25 \times 10^{-11}$ H	resonator self inductance
L_q	$= 1.38 \times 10^{-11}$ H	qubit self inductance
M_{rq}	$= 6.75 \times 10^{-12}$ H	mutual inductance between resonator and qubit
$\omega_q/2\pi$	$= 6$ GHz	qubit energy level splitting
Φ	$= 2.37 \times 10^{-12}$ Wb	flux through the resonator
T_q	$= 100$ mK	temperature of qubit system
$I_{q\text{max}}$	$= 75\,\mu$A	maximum current in qubit
I_{q0}	$= 3.5 \times 10^{-14}$ A	minimum current in qubit
$I_{r\text{max}}$	$= 48\,\mu$A	maximum current in resonator
τ_{exp}	$= 87.8\,\mu$s	time for one complete prepare / evolve / measure run
τ_c	$= 70\,\mu$s	coherence time of the qubit
T_1	$= 70\,\mu$s	qubit T_1 coherence time
T_2	$= 70\,\mu$s	qubit T_2 coherence time
Γ_{gas}	$= 2.7 \times 10^{-8}$ Hz	resonator amplitude damping rate due to background gas collisions
Γ_{eddy}	$= 8.1 \times 10^{-19}$ Hz	resonator amplitude damping rate due to induced eddy current losses
Γ_{rad}	$= 3.3 \times 10^{-22}$ Hz	resonator amplitude damping rate due to magnetic dipole radiation
